# *CYP2D6* Genotyping for Optimization of Tamoxifen Therapy in Indonesian Women with ER+ Breast Cancer

**DOI:** 10.3390/jpm15030093

**Published:** 2025-02-28

**Authors:** Baitha Palanggatan Maggadani, Kathleen Irena Junusmin, Fatma Aldila, Jessica Audrienna, Bijak Rabbani, Yusuf Maulana, Sabrina Gabriel Tanu, Gabriella Gabriella, Margareta Amelia, Faustina Audrey Agatha, Marco Wijaya, Stevany Tiurma Sormin, Caroline Mahendra, Levana Laksmicitra Sani, Astrid Irwanto, Alexandre Chan, Harmita Harmita, Yahdiana Harahap, Samuel Johny Haryono

**Affiliations:** 1 Faculty of Pharmacy, University of Indonesia, Jakarta 16424, Indonesia; baitha.p@farmasi.ui.ac.id (B.P.M.);; 2 Nalagenetics Pte Ltd., Bukit Merah, Singapore 169204, Singapore; 3 SJH Initiatives, MRCCC Siloam Hospitals Semanggi, Jakarta 12930, Indonesiamarcowijaya51@gmail.com (M.W.);; 4 Department of Pharmacy, Faculty of Science, National University of Singapore, Singapore 119077, Singapore; 5 Department of Clinical Pharmacy Practice, School of Pharmacy & Pharmaceutical Sciences, University of California, Irvine, CA 92697, USA; a.chan@uci.edu; 6 Faculty of Military Pharmacy, Indonesia Defense University, Bogor 16810, Indonesia

**Keywords:** *CYP2D6* polymorphisms, endoxifen, pharmacogenomics, dose adjustment, allele frequency, phenotype distribution, metabolizer profile

## Abstract

**Background:** Certain *CYP2D6* genotypes are linked to a lower efficacy of tamoxifen therapy. This study aimed to observe *CYP2D6* polymorphisms and examine the impact of *CYP2D6* genotyping among tamoxifen-treated breast cancer patients in Indonesia. **Methods:** 150 breast cancer participants were recruited. Buccal swab samples were collected; gDNA was extracted and genotyped using the qPCR method. Blood samples were collected, and measurement of tamoxifen metabolite levels was performed using UPLC-MS/MS. **Results:** 43.3% (n = 65) of participants were IMs. *10 was the most common haplotype (n = 89, 29.7%), followed by *36 (n = 73, 29.7%), making *10/*36 the most common diplotype (n = 34, 22.7%) in this study. The difference in endoxifen levels between the NM and IM-PM groups at baseline was statistically significant (*p* ≤ 0.001). A dose increase in tamoxifen to 40 mg daily successfully increased endoxifen levels in IMs to a similar level with NMs at baseline (*p* > 0.05) without exposing IMs to serious side effects. No statistically significant differences were observed between the 20mg group and the 40 mg group on the adjusted OS (*p* > 0.05) and the adjusted PFS (*p* > 0.05). **Conclusions:** Our study observed a considerably high proportion of *CYP2D6* IMs. The dose adjustment of tamoxifen was proven to significantly and safely improve the level of endoxifen and survival.

## 1. Introduction

Estrogen-receptor-positive (ER+) breast cancers account for about 80% of invasive breast cancers. Furthermore, the tumor cells’ levels of estrogen receptor (ER) expression are closely linked to the therapeutic effects of endocrine medications that target ER [1]. Hormone-receptor-positive breast cancer is associated with clinically less aggressive features and a better prognosis because of the benefits from currently available endocrine therapy [2]. Tamoxifen is the current standard of care for ER+ breast cancer adjuvant therapy in premenopausal women [3]. The drug has been proven effective in reducing the number of recurrences, especially in pre-menopausal women. In Indonesia’s National Cancer Management Guidelines for Breast cancer, tamoxifen is the first-line treatment for hormonal therapy [4]. With a total of breast cancer incidence at approximately 58,000 in 2018 [5], it is implied that the prescription of this drug is prevalent in Indonesia.

Tamoxifen is a prodrug that needs to be metabolized by the cytochrome P450 2D6 (CYP2D6) enzyme into endoxifen to be active. However, the *CYP2D6* gene has many variants that may result in non-functional or reduced function alleles. Some of the variants cause reduced activity, while some others cause complete loss of function. With regards to *CYP2D6* gene genetic polymorphism, an individual is usually classified to one of four groups: extensive or normal metabolizer (NM), ultrarapid metabolizer (UM), intermediate metabolizer (IM), and poor metabolizer (PM), depending on how many reducing and/or loss of function alleles the individual carries [6]. *CYP* polymorphism prevalence varies significantly among ethnic groups and is a significant factor in inter-individual and inter-ethnic variations in medication metabolism and response. For instance, only 2–3% of Europeans have a *CYP2D6* UM profile, whereas 20–29% of people in East Africa have one [7]. On the other hand, around 43% of Asian populations have the reduced function allele CYP2D6*10, indicating IM profile [8].

Endoxifen, the main metabolite of tamoxifen, is formed predominantly by *CYP2D6* from N-desmethyltamoxifen and plays an important role to the overall anticancer effect as it has a 100-fold higher affinity for estrogen receptor than tamoxifen itself [9]. Endoxifen serum threshold value has been discovered to be associated with breast cancer recurrence risk [10]. It has been shown that endoxifen serum levels above 5.9 ng/mL are associated with a 30% lower chance of having recurrence of breast cancer [11]. Another study has also shown that individual variability of *CYP2D6* gene contributed 53% towards the ratio of N-desmethyltamoxifen and endoxifen, while genetic factors from the other *CYP* genes and non-genetic factors (i.e., age, BMI) combined contributed to only 2.8% [12]. However, data on the distribution of *CYP2D6* genotypes in ER+ breast cancer patients in Indonesia are missing.

In addition, studies on the clinical outcomes of *CYP2D6* polymorphisms still showed varying results. A 2017 meta-analysis from various Asian countries has found that CYP2D6*10 polymorphisms affect the effectiveness of tamoxifen treatment, which is frequently observed in the Chinese population. Another study has also demonstrated similar concepts when patients from a Chinese population taking tamoxifen with *CYP2D6* *10 T/T experienced a reducing effect of the respective drug [13]. In contrast, two studies involving post-menopausal women taking tamoxifen failed to show any significance between endoxifen levels or *CYP2D6* genotypes and clinical outcomes [6].

This study aimed to observe *CYP2D6* polymorphisms and examine the impact of *CYP2D6* genotyping among tamoxifen-treated breast cancer patients in Indonesia.

## 2. Materials and Methods

### 2.1. Study Design and Ethical Consideration

This study is a single-arm, open-label, prospective study. A randomized controlled trial was not performed as it does not allow the researchers to allocate benefits of genetic testing to a wider targeted population beyond the treatment group. Blinding was not possible because participants received clinical recommendations based on their genetic testing results. Blinding was also not beneficial because the primary outcome measures were objective. The trial was registered at ClinicalTrials.gov with identifier NCT04312347.

Institutional Review Board (IRB) approval was granted by MRCCC Siloam Hospitals Semanggi Ethics Review Committee (Jakarta, Indonesia) under IRB Reference Number 001/EA/KEPKK/RSMRCCC/V/2019. A protocol amendment was submitted to include survival analysis, and this was approved under IRB Reference Number 004/EA/KEPKK/RSMRCCC/X/2020.

### 2.2. Recruitment

Participants were recruited at the Breast Cancer Care Alliance (BCCA) Clinic in MRCCC Siloam Hospitals Semanggi Jakarta, Indonesia. Breast cancer patients who fulfilled the inclusion criteria obtained information about the study and were offered the opportunity to participate. A written informed consent was obtained before they were enrolled in the study.

### 2.3. Eligibility Criteria

The inclusion criteria of this study were (1) female patients who had been diagnosed with ER+ breast cancer and (2) had been consuming tamoxifen as their adjuvant hormonalc therapy for at least eight weeks at the time of recruitment. The patients were excluded if they were (1) under 18 years old or (2) declined to participate in the study.

### 2.4. Sample Size Calculations

To determine the minimum sample size, a power calculation was conducted using GPower software version 3.1.9.6. We estimated the sample size utilizing a *t*-test for two independent means with an effect size set at 0.7. The effect size was determined based on similar studies that show high effect sizes [14,15]. The calculation yielded a sample size requirement of 44 samples for each of the cases and control groups, totaling 88 samples, under the assumption of a two-tailed test with an alpha of 5% and a power of 90%. Nonetheless, we opted to collect a larger sample size than calculated to mitigate potential errors and account for anticipated dropouts.

### 2.5. Data Collection

At recruitment, we collected demographic data, including date of birth, weight, height, cancer stage, menopausal status, and ethnicity. The data were collected using a standardized form (See File S1). Research members responsible for recruitment verbally gathered the data from participants and wrote the data on the designated form.

### 2.6. Intervention

The intervention received by participants was genotype-based therapeutic recommendations. The recommendations given were based on the treating oncologists’ clinical judgement and existing guidelines. Several guidelines in consideration include guidelines from the Clinical Pharmacogenetics Implementation Consortium (CPIC), the National Surgical Oncologist Organization and Ministry of Health in Indonesia [4], the National Comprehensive Cancer Network [3], and the British Columbia Cancer Agency [16].

When the genetic testing results were available, participants would be informed and invited to attend a follow-up consultation. The treating oncologists would first explain the genetic test results then give appropriate therapeutic recommendations. Participants classified as UMs or NMs remained on the standard dose of tamoxifen [17]. Meanwhile, those who were IMs or PMs were recommended by treating oncologists to increase their tamoxifen dose to 40 mg. However, individuals who were clinically unfit for a tamoxifen dosage increment, for example those who were entering their post-menopausal period, were recommended to switch to an aromatase inhibitor.

### 2.7. Outcomes

The primary outcomes of the study were the distribution of *CYP2D6* genotypes and phenotypes among the Indonesian population and the impact of personalized dosing of tamoxifen on an endoxifen level.

Buccal swab samples were first collected from the participants for *CYP2D6* genotyping using the ORAcollect•DNA OCR-100 (DNA Genotek Inc, Ottawa, ON, Canada) swab. Genomic DNA (gDNA) extraction was performed from the buccal swab samples using the Monarch Genomic DNA Purification Kit (NEB #T3010) (New England Biolabs, United Kingdom) following the manufacturer’s instructions. The concentration of gDNA extracts was quantified using BioDrop spectrophotometer (Harvard Bioscience, United Kingdom). Acceptance criteria to further process the gDNA extracts for genotyping included: (1) total DNA yield ≥ 500 ng, (2) A260/280 ratio ≥ 1.75, and (3) A260/230 ratio ≥ 1.75. gDNA extracts were diluted to 2 ng/uL and added as a template for genotyping.

The samples were run on the Bio-Rad CFX96 Touch^TM^ Real-Time PCR Detection System and genotyped by using Nala PGx Core^TM^ qPCR (NalaGenetics, Singapore), a lab-developed test genotyping panel consisting of four pharmacogenes: *CYP2D6*, *CYP2C19*, *CYP2C9,* and *SLCO1B1* [18]. *CYP2D6* haplotypes, diplotypes, and phenotypes were inferred by Nala Clinical Decision Support^TM^, which is a class A medical device (Health Sciences Authority, Singapore) compatible with Nala PGx Core^TM^ qPCR output. In addition to the total copy number of intron 2 and a detection for the presence of exon 9 conversion, a number of *CYP2D6* star alleles were genotyped in this test. The list of star alleles genotyped with their corresponding rs identifiers are listed in Table 1.

Peripheral whole blood samples were collected to perform the measurement of the metabolite level. A finger-prick blood sample was obtained using the volumetric absorptive microsampling (VAMS) technique. VAMS extraction was performed in methanol by a sonication-assisted extraction method for 25 min after 2 h of VAMS drying. Separation was carried out using an Acquity UPLC BEH C18 column (2.1 × 100 mm; 1.7 µm (Waters, Milford, USA)), with a flow rate of 0.2 mL/minute, and the mobile phase gradient of formic acid 0.1% combined with formic acid 0.1% in acetonitrile for 5 min. The UPLC-MS/MS Waters Xevo TQD Triple Quadrupole with MassLynx Software controller (Waters, Milford, USA) was employed in metabolite measurement. Mass detection was carried out utilizing Triple Quadrupole (TQD) with multiple reaction monitoring (MRM) analysis modes and an electrospray ionization source using the positive mode. The method was developed in the Bioavailability and Bioequivalence Laboratory of Universitas Indonesia and validated according to FDA and EMA guidelines [15]. The multiple reaction monitoring (MRM) values were set at *m*/*z* 372.28 > 72.22 for tamoxifen; 374.29 > 58.22 for endoxifen; 388.29 > 72.19 for 4-hydroxytamoxifen; 358.22 > 58.09 for and N-desmethyltamoxifen; and 260.20 > 116.20 for propranolol as the internal standard. The level of tamoxifen metabolites, including tamoxifen, endoxifen, 4-hydroxytamoxifen, N-desmethyltamoxifen, were assessed twice. First, the metabolite level was measured at baseline among all study participants. This was to confirm if IMs and PMs indeed would have a lower endoxifen level compared to NMs at a standard dosage of tamoxifen secondary to suboptimal drug metabolism. Subsequently, the level of metabolites was reassessed 8 weeks post dose adjustment among those recommended to increase their tamoxifen dose to 40 mg daily. The reassessment was performed to test the notion that tamoxifen dose adjustment could make the endoxifen levels of IMs and PMs comparable to NMs at baseline.

The secondary outcomes of the study were the adverse drug reactions reported by study participants and their survival over 3 years.

The presence of side effects, such as endocrine-therapy-related symptoms, is considered a predictor of tamoxifen non-adherence [19]. In addition, more severe side effects might be observable in the case of tamoxifen dosage increase. Therefore, the symptoms were recorded using the FACT-ES questionnaire [20]. A Google form containing the FACT-ES questionnaire was circulated via WhatsApp message to all study participants 8 weeks after the follow up consultation with treating oncologists. To increase the response rate, the same message was sent again to all participants 9 and 10 weeks after the consultation.

In addition, to assess the clinical outcomes of the personalized dosing of tamoxifen, participants who remained taking tamoxifen, either at the standard dose of 20 mg or at an adjusted dose of 40 mg daily, were followed up for a minimum of three years for estimation of overall survival (OS) and progression-free survival (PFS). Three years was deemed an appropriate observation period for survival analysis as this study included participants regardless of their disease stage or age. The survival rate of breast cancer, for example, tends to fall off significantly as disease progression is observed. Breast cancer patients in Indonesia at the stage of IIA have 100% 3-year OS rate, while the 3-year OS rate for the stage IV patients is only 30% [21].

The OS rate was calculated as the percentage of study participants in each group who were still alive within the 3-year follow-up period, and the PFS rate was determined by calculating the percentage of study participants in each group whose condition did not worsen until the 3-year follow-up period ends. The analysis for hazard ratio was determined using R version 4.2.2. A simple Kaplan–Meier chart was also created using Microsoft Excel version 2501 to visualize the survival curve. Meanwhile, the incidence of local recurrence and distant metastasis was monitored to determine the disease progression. Mammography, Breast USG results, CA 15-3 level, and other medical imaging results, where available, were used to help the assessment.

### 2.8. Data and Statistical Analysis

The statistical analysis was performed using Microsoft^®^ Excel^®^ for Microsoft 365 and R version 4.2.3.

On the results of genotyping, deviation from Hardy–Weinberg equilibrium was performed on the haplotype frequencies using the chi-square statistical test, where Bonferroni correction was applied to determine the *p*-value threshold for significant deviation.

A comparison test using *t*-test was performed to see if metabolite level distribution at baseline was statistically different across all phenotypes. The comparison of metabolite levels before and after dose adjustment for those receiving a higher dose of tamoxifen was carried out using a *t*-test, and the same was used to compare the distribution of metabolite levels in IMs post-dose adjustment against NMs at baseline.

While assessing the difference in endoxifen levels before and after the intervention, we performed a multivariate regression with several covariates. This was carried out to confirm if BMI and age were associated with endoxifen level at baseline, as stated in several studies. Madlensky et al., for example, found that BMI and age might also be associated with endoxifen level [22]. However, a study by Braal et al. (2022) identified an association between *CYP2D6* phenotype and endoxifen level that was independent from individual BMI and age [10].

The chi-square test was also performed to investigate the endocrine-therapy-related symptoms experienced by participants recommended to take 40 mg tamoxifen daily compared to those recommended to continue taking 20 mg tamoxifen daily.

The logrank test was conducted to compare the survival between the two groups according to the intention-to-treat (ITT) principle. Both age and disease stage during recruitment were considered potential confounders of this analysis [23,24]. Identification and adjustment of confounders, if any, were performed using multivariate regression.

## 3. Results

### 3.1. Recruitment Outcomes

**Figure 1:**
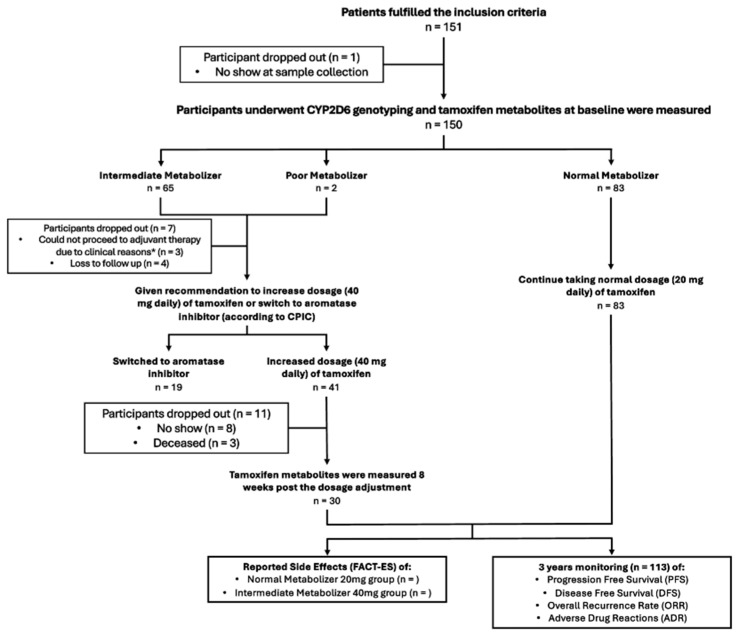
Research flow diagram.

Breast cancer patients visiting the BCCA Clinic in MRCCC Siloam Hospital, Semanggi, between October 2019 and April 2021 who met the inclusion criteria were offered participation in the study. However, only 151 of them consented to participate in the study. Throughout the course of the study, a total of 19 participants dropped out, making the final number of study participants included in the primary outcome analysis 113. The research flow diagram is available as Figure 1.

### 3.2. Demographics of Study Participants

Most of the participants were aged between 40 to 49 years old (n = 88, 58.7%) and fell into the normal BMI group (n = 88, 58.7%). At the time of recruitment, most of them were in their early stage of cancer, with 24.0% (n = 36) and 32.0% (n = 48) of them in Stage I and Stage IIA, respectively. Postmenopausal participants accounted for 64.0% of the study population (n = 96). Chinese Indonesian individuals (n = 50, 33.3%) made up the majority of the study population, followed by Javanese (n = 38, 25.3%). The demographics of the study participants are presented in Table 2.

### 3.3. Distribution of *CYP2D6* Haplotypes, Diplotypes, and Phenotypes

**Figure 2:**
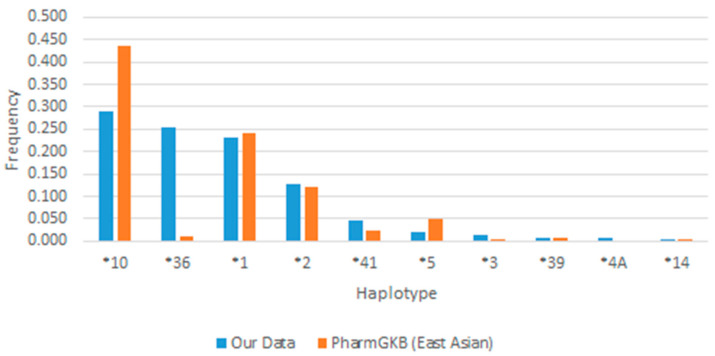
Distribution of haplotype frequencies (N = 300). The study exhibits a similar distribution of haplotype frequencies compared to those from the East Asian population dataset of PharmGKB. The * within this figure stands for the haplotype observed in the study.

All haplotypes observed were in Hardy–Weinberg equilibrium (*p* > 0.005). As shown in Figure 2, *10 was found to be the most abundant haplotype in the study population (n = 89, 29.7%), followed by *36 (n = 73, 24.3%). The reference haplotype, *1, was observed with a frequency of 22.7% (n = 6).

Our study found *10/*36 (n = 34, 22.7%) to be the most abundant diplotype in the study population, followed by *1/*36 (n = 19, 12.7%). The other diplotypes that were observed in this study with diplotype frequencies ranging between 0.1 and 0.05 were *2/*10 (n = 15, 10%), *1/*1 (n = 13, 8.7%), *2/*36 (n = 12, 8.0%), *1/*10 (n = 12, 8.0%), and *10/*10 (n = 9, 6.0%). Among these, copy number duplications (>=3) were observed in 17 individuals. Table 3 shows the *CYP2D6* diplotype frequencies.

**Figure 3:**
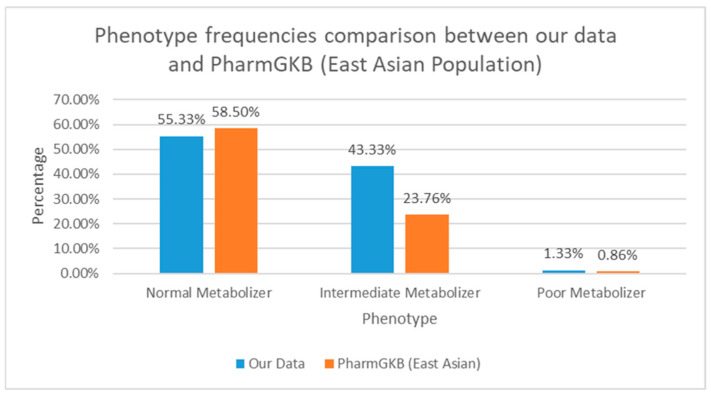
Distribution of phenotype frequencies (N = 150). Consistencies with the East Asian population of PharmGKB dataset were observed for frequencies of NMs and PMs.

**Figure 4:**
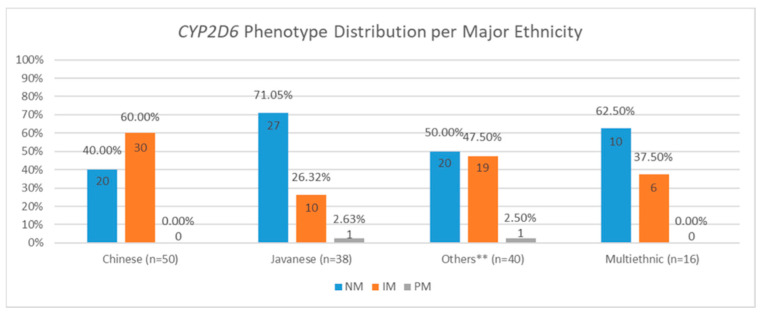
Distribution of phenotype frequencies based on ethnicity (N = 144). NMs were more frequent among the Javanese participants, while most of the IMs were the Chinese participants. ** The data of other ethnicities were combined for simplified visualization and the data do not represent 6 participants with unknown ethnicity.

The majority of participants belong to the NM group (n = 83, 55.33%), followed by IM group (n = 65, 43.33%) as the second largest group. There were two study participants classified as PM (1.33%) and no UMs were observed in this cohort. The distribution of *CYP2D6* phenotypes among all study participants is shown by Figure 3. In addition, Figure 4 showed the distribution of *CYP2D6* phenotypes based on ethnicity. It is visible that most of the Chinese Indonesian participants were IMs (n = 30, 60.00%), and Javanese study participants were mostly NMs (n = 27, 71.05%).

### 3.4. Impacts of Personalized Dosing to Levels of Tamoxifen Metabolites

PM and IM participants were grouped together for the statistical analysis of the association between phenotypes and tamoxifen metabolites at baseline. This is because there were only two participants classified as PM. Additionally, both PM and IM participants received the same recommendation from CPIC, that is, to increase their tamoxifen dosage. Confounding factors were not identified as the associations between BMI and age with endoxifen level were not statistically significant, with *p* > 0.05. The statistical analysis at baseline showed that the endoxifen level of the PMs-IMs was significantly lower than NM (*p* < 0.001). The rest of the metabolites did not show any statistically significant distribution among phenotypes (*p* > 0.05 for tamoxifen, 4-hydroxytamoxifen, and N-desmethyltamoxifen, respectively). A summary of the level of metabolites at baseline in relation to *CYP2D6* phenotypes is shown in Table 4.

A follow-up consultation was individually scheduled for each participant to inform treatment adjustment, where relevant. There were 83 NMs who were advised to continue taking the standard dose of 20 mg daily. Meanwhile, there were 65 IMs and 2 PMs who were given a recommendation to adjust their tamoxifen dose to 40 mg daily. However, a total of 17 IMs and 2 PMs were deemed clinically unfit for tamoxifen dosage increase and recommended to switch to aromatase inhibitor. Individuals switching to aromatase inhibitors were not followed up further for assessment of metabolite levels changes, side effect monitoring, and survival analysis as tamoxifen is this study’s drug of interest. Furthermore, eight participants from the 40 mg group did not show up for further assessment of the treatment adjustment. Three IMs were reported deceased during the treatment adjustment period. This makes a total of 131 NMs (n = 83, 63.4%) and IMs (n = 48, 36.6%) included in the further assessment after the treatment adjustment.

**Figure 5:**
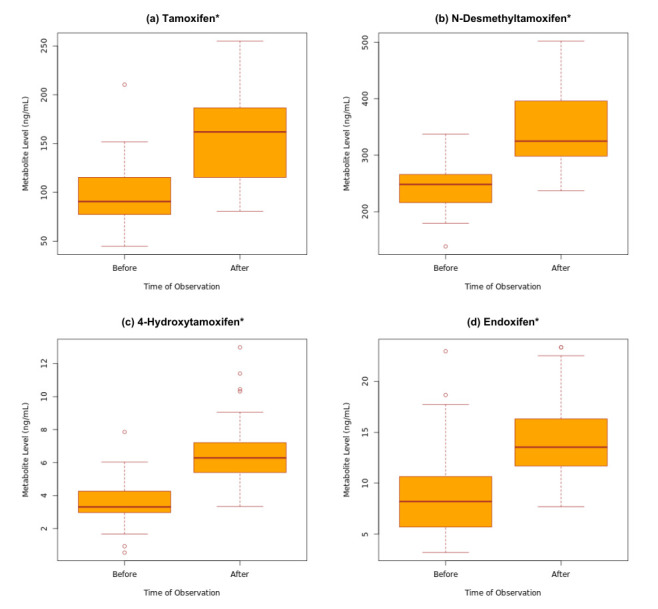
Metabolite levels before and after dose adjustment for IMs. (**a**) Tamoxifen (*p* < 0.001), (**b**) endoxifen (*p* < 0.001), (**c**) 4-hydroxytamoxifen (*p* < 0.001), (**d**) N-desmethyltamoxifen (*p* < 0.001). * Statistically significant differences were observed between metabolites before and after dose adjustment, n = 30.

The metabolite levels in IMs (n = 30, 26.50%) post dose adjustment to 40 mg was compared against NMs (n = 83, 73.50%) taking 20 mg at baseline to assess if the treatment adjustment could inform an optimum, personalized dose of tamoxifen. Figure 5 showed the significant increase in the level of all metabolites from baseline to post dose adjustment. In addition, there was not a statistically significant difference in the level of endoxifen between NMs at 20 mg and IMs at 40 mg (*p* > 0.05), as seen in Table 5.

### 3.5. Reported Side Effects 8 Weeks After Post-Test Consultation with Treating Oncologists

The FACT-ES survey was completed by 53 study participants. As shown in Table 6, there were 31 of 83 NMs (37.3%) and 26 of 48 IMs (54.2%) who completed their 8-week follow up period and reported symptoms related to endocrine therapy. The most reported symptoms by IMs were mood swings (n = 17, 65.4%), weight gain (n = 17, 65.4%), and irritability (n = 16, 61.5%). Meanwhile, the most reported side effects in the NM group were mood swings (n = 23, 74.2%), pain in joints (n = 21, 67.7%), lost interest in sex (n = 20, 64.5%), and weight gain (n = 20, 64.5%). A chi-square test performed concerning symptoms reported by IM participants taking tamoxifen 40 mg daily and NM participants taking tamoxifen 20 mg daily resulted in only two symptoms (pain or discomfort during intercourse and lost interest in sex) having statistical significance between the 40 mg and the 20 mg groups (*p* < 0.001 and <0.05, respectively). Serious side effects including thrombosis, endometriosis, and endometrial cancer were also not observed in all groups within 8 weeks.

### 3.6. Survival Analysis

A total of 113 participants were enrolled in the survival study. The period of observation was between 20 September 2019 and 6 April 2024. After 3 years, 20 participants were lost to follow up, with 4 belonging to the 40 mg group and the remaining 16 participants coming from the 20 mg group. A total of 12 participants were reported to be deceased, 2 of them were participants from the 40 mg group, and the remaining 10 were those from the 20 mg group. In addition, 24 events of progression were observed, contributed by 7 participants from the 40 mg group and 17 participants from the 20 mg group.

**Figure 6:**
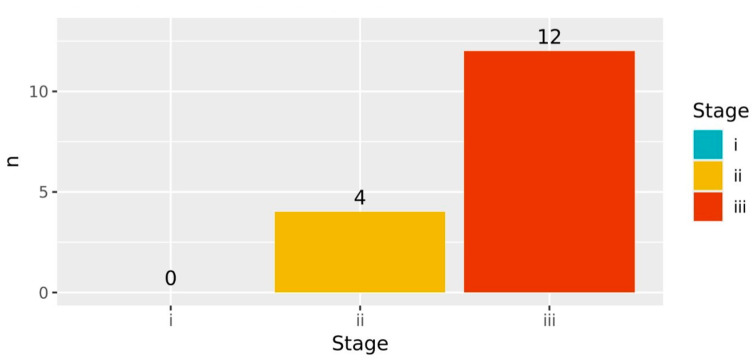
Number of deceased participants. Most of the death events were reported among those recruited at Stage III.

**Figure 7:**
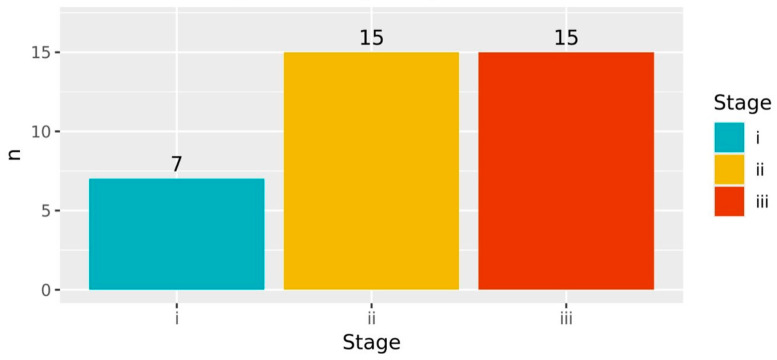
Number of participants surviving but experiencing progression. The numbers of participants surviving with progression were observed to be higher among those recruited at Stage II and III.

Both age and stage were suspected to be potential confounders of survival analysis. However, the statical analysis found no statistically significant association between age and both OS (*p* > 0.05) and PFS (*p* > 0.05). Statistically significant associations were indeed observed between disease stage at recruitment with both OS (*p* < 0.001) and PFS (*p* < 0.05), as shown by Figure 6 and Figure 7. Figure 6 shows that the deceased participants were mostly individuals who were at Stage III at recruitment. In addition, Figure 7 shows that the participants who survived but experienced progression were dominated by individuals who were either at Stage II or Stage IIII at recruitment. Accordingly, the disease stage was treated as a covariate of the survival analysis.

**Figure 8:**
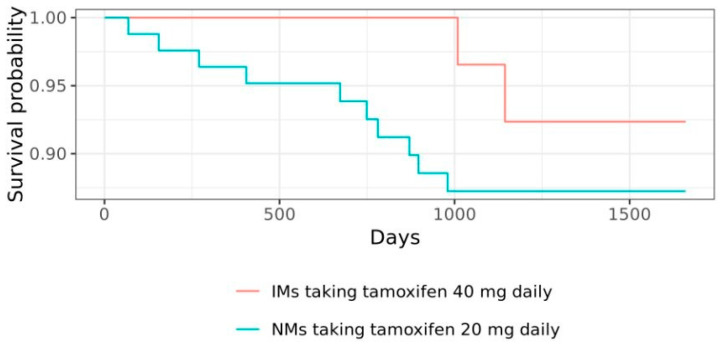
Kaplan–Meier chart for OS after 3-year monitoring. There was no significant difference between the OS of IMs taking tamoxifen 40 mg daily and NMs taking tamoxifen 20 mg daily.

**Figure 9:**
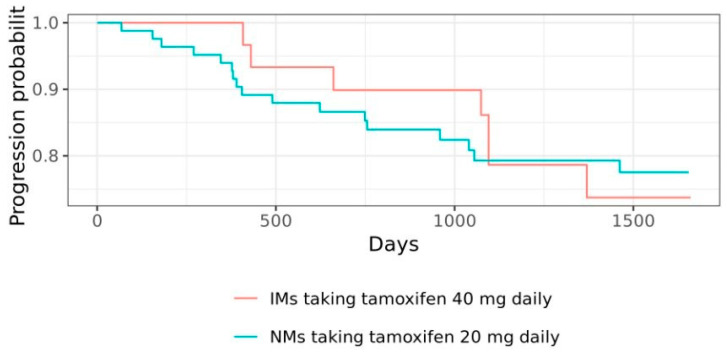
Kaplan–Meier chart for PFS after 3-year monitoring. No significant difference was observed in the PFS of IMs taking tamoxifen 40 mg daily and NMs taking tamoxifen 20 mg daily.

Without considering disease stage as a confounding factor, it is found that OS rate was 88% (37/42) in the 40 mg group and 93% (77/83) in the 20 mg group (HR 2.02, 95% CI 0.44 to 9.22; *p* > 0.05). Meanwhile, the PFS rate was 77% (32/42) in the 40 mg group and 80% (66/83) in the 20 mg group (HR 0.97, 95% CI 0.40 to 2.35; *p* > 0.05). After adjusting for disease stage with the reference population from Widiana et al. (2020), the OS rate was 78% (33/42) and 77% (64/83) for the 40 mg and 20 mg group, respectively (HR 2.05, 95% CI 0.45 to 9.39; *p* > 0.05). Meanwhile, the adjusted PFS rate is 54% (23/42) at 40 mg and 72% (60/83) in the 20 mg group (HR 0.91, 95% CI 0.37 to 2.19; *p* > 0.05). Figure 8 and Figure 9 are the Kaplan–Meier charts showing no significant difference in the OS and the PFS between PM/IM participants given 40 mg tamoxifen and NM participants given 20 mg tamoxifen daily. The most prevalent disease progression was identified to be lung metastasis, which was seen in nine study participants in the 20 mg group and two in the 40 mg group, followed by bone metastasis (six in the 20 mg group and one in the 40 mg group) and liver metastasis (two in the 20 mg group and two in the 40 mg group).

## 4. Discussion

The study has validated the benefit of pharmacokinetics and pharmacogenomics investigation to reach optimum therapy with tamoxifen. To the extension of our knowledge, this is the first study aiming to evaluate the benefit of tamoxifen metabolite level measurement and *CYP2D6* genotyping for personalized dosage of tamoxifen.

In our study, *10 was the most common haplotype, followed by *36. The distribution of *10 was lower and the distribution of *36 was much higher compared to the East Asian population dataset available on the PharmGKB, as shown by Figure 2. Despite the non-deviation from the Hardy–Weinberg equilibrium, the reported star allele frequencies were observed to have differences compared to PharmGKB East Asian dataset, which could be contributed to by selection bias from inclusion of only breast cancer women patients. Our results are consistent with those of Yusuf et al. in Singapore, who found that Chinese and Malay ethnicities primarily exhibited *36 and *10 alleles [25]. Additionally, a study conducted in the Han Chinese population also recorded relatively high frequencies of CYP2D6*10 and CYP2D6*36 of 65.84% and 39.10%, respectively [26]. In addition, *10/*36 was the most common diplotype. The *10/*36 translates as the IM phenotype, which suggested that *36 may play an important role in constructing IM phenotype profiles among study participants.

The majority of the study participants were NMs with *CYP2D6* (55.83%), followed by IMs (40.7%). The proportions are within the range of the current known global prevalence, which is between 43 and 67% for NMs and 10 and 44% for IMs. A similar study conducted in the Vietnamese population showed a relatively high frequency of the population with allele combinations that would predict an IM phenotype compared to the global prevalence (50%), implying that the East Asian population may have a relatively higher frequency of IMs [27]. The high proportion of participations below in the IM group indicates that the Indonesian population might be at higher risk of experiencing ineffectiveness of tamoxifen therapy. In addition, the proportion of IMs in this study (40.7%) was similar to those of a similar study conducted on the Han Chinese population, which was 45.4% [28]. This corresponds to our study where the majority of the participants were Chinese Indonesians (33.3%), and they were mostly IMs.

Our study found a significant difference in the endoxifen levels between NMs and IMs and PMs, where lower endoxifen levels may indicate lower efficacy of tamoxifen in preventing recurrence. The average value of endoxifen level among IMs observed in this study, that is, 9.6 ng/mL, was higher compared to the study conducted by Madlensky et al. (2011), that is, 8.1 ng/mL [22]. However, a study conducted in the Swedish population found a range in endoxifen levels between 2.3 and 16 ng/mL [29], while another study conducted in Zimbabwe displayed a range between 3.88 and 8.49 ng/mL with a median of 4.78 ng/mL [30]. These suggested that studies conducted with similar interventions may result in different ranges of metabolite levels. Differences in metabolite level range may be due to the measurement or sampling using different techniques. While other studies mostly measure metabolite levels from plasma or serum, our measurement used peripheral whole blood using the VAMS technique due to its effectiveness and ease of use, which allows non-medical professionals to perform the sampling procedure. This sampling technique also provides a more stable condition of the blood samples for storage and transport purposes [15].

Our study has shown that IM participants who received 40 mg of tamoxifen daily all experienced a significant increase across all metabolite levels, primarily the level of endoxifen. This suggested that increasing tamoxifen intake can elevate endoxifen levels as expected and may be effective in ensuring the therapeutic effect of tamoxifen. The distribution of endoxifen level in IMs post dose adjustment were similar to the endoxifen level in NMs at the baseline, suggesting that increasing tamoxifen dosage to 40 mg daily for IM participants had successfully let IM participants reach the expected endoxifen levels as observed in NMs. The findings of this study are supported by Khalaj et al. (2020), where 134 ER+ breast cancer patients were included in a similar prospective clinical trial. The study participants were graded based on their *CYP2D6* activity score (AS). Those with an AS of 1 (NMs) were assigned a tamoxifen dose of 30 mg/day, while those with an AS of 0–0.5 (PMs-IMs) received 40 mg/day. After 8 months of treatment, endoxifen concentrations were remeasured. The study observed a significant increase in median endoxifen levels, rising from 11.9 nM at baseline to 23.5 nM post-adjustment. These adjustments led to endoxifen concentrations in patients with lower AS becoming comparable to those with AS > 1 receiving the standard 20 mg/day dose [6].

Pain or discomfort during intercourse and lost interest in sex were the only two symptoms with a statistically significant difference between the 40 mg and the 20 mg groups. In addition, dose escalation up to 40 mg daily did not expose serious side effects to study participants. Although the CPIC guideline recommended the first course of action to be switching to aromatase inhibitors, our study demonstrated that taking tamoxifen at 40 mg daily is shown to be as safe as taking tamoxifen at 20 mg daily. Gynecological side effects similar to menopausal symptoms, such as hot flushes, vaginal dryness, and endometriosis, were commonly reported by individuals taking tamoxifen regardless of dose [31,32,33]. Other studies that have tried to observe tamoxifen side effects occurring in patients with dose increase also concluded that increasing tamoxifen dose did not result in toxicity or short-term increase in side effects [10,34]. Additionally, despite some of the IM respondents in this study who received a dose increase reporting experiencing hot flashes, no respondents reported dismissing tamoxifen intake due to the symptoms, which indicated nonadherence secondary to the treatment side effects. These findings were expected to serve as an early scientific basis to assess and adjust ER+ breast cancer adjuvant therapy guidelines, where physicians could be given options when determining the most suitable adjuvant treatment for each individual patient.

The study did not observe a significant difference in the survival and disease progression of participants classified as NMs and maintained their tamoxifen dosage at 20 mg daily of tamoxifen compared to those classified as PMs or IMs and increased their tamoxifen dosage to 40 mg daily, suggesting that *CYP2D6* genotyping has the potential to make the survival rate of the two groups comparable. This is imperative since *CYP2D6* polymorphisms were considered one of the prognostic markers of breast cancer survival. The study by Lan et al. (2018) among the Chinese Han population, for example, found that individuals taking tamoxifen and carrying specific variants of *CYP2D6* genes, such as CYP2D6*10 T/T, were observed to have a lower disease-free progression compared to those CYP2D6*10 C/C or C/T individuals [35]. Some studies also suggested that some of the *10 alleles increase the risk of breast cancer recurrence for those taking tamoxifen as adjuvant therapy [36].

We identify several limitations of this study. First, the majority of the study participants were the Chinese Indonesian participants, and they were predominantly IMs. Meanwhile, those of the Javanese ethnicity were commonly NMs. We are aware that the genetic profile of this group might not represent the genetic profile of the majority of the Indonesian population. Ethnicity also affects the proportion of phenotype profiles observed. For example, a higher proportion of NMs is usually observed in the Caucasians though the proportions are slightly varied depending on the geographical location where the studies were conducted [34,37,38]. Second, the observation was time limited. The study participants were only monitored for 8 weeks post follow-up consultation to assess changes in endoxifen level and immediate side effects. The relatively short period of follow-up duration might have led us to underestimate changes in metabolite level or occurrence of side effects that might not immediately show up after therapy adjustment. The Irvin et al. study, for example, monitored the participants for 4 months before performing data analysis [39]. Serious side effects, such as thrombosis, endometriosis, and endometrial cancer [40,41,42], might also not develop immediately. Similarly, the cohort was only followed up for a minimum of 3 years for the survival analysis. Monitoring the cohort for a longer period might be useful for a more accurate evaluation of survival considering the cohort size. In addition, side effects were self-reported by the study participants using the FACT-ES questionnaire, which could lead to recall and response biases. Finally, due to the adoption of ITT principle, there is a possibility that study participants change their medical institutions during the 3-year follow up period or decide to voluntarily draw back from the research. Some data points might also be missing due to the lack of routine checkups and screening from participants as the study was initiated during the COVID-19 pandemic. Given Indonesia’s diverse ethnic groups, future research should aim to include many more representatives of other ethnic groups. Future research could also investigate the feasibility of implementing endoxifen monitoring and *CYP2D6* phenotyping in clinical settings for breast cancer patients, followed by the cost-effectiveness evaluation.

## 5. Conclusions

Our study has observed a considerably high proportion of *CYP2D6* IMs among the Indonesian women with ER+ breast cancer consuming tamoxifen. The study also found a significant difference in the endoxifen levels between NMs and IMs and PMs when all of them took 20 mg of tamoxifen daily. A dose adjustment of tamoxifen was proven to significantly improve the level of endoxifen and survival. These findings indicated the benefit of pharmacokinetics and pharmacogenomics investigation to optimize tamoxifen treatment.

## Figures and Tables

**Table 1:** List of star alleles genotyped with their corresponding rs identifiers.

rs Identifiers	*CYP2D6* Star Alleles
rs35742686	*3
rs59421388	*29, *70, *109, *149, *155, *156, *157, *165, *171, *175
rs3892097	*4
rs5030656	*9, *109, *115
rs72549352	*21
rs5030655	*6
rs28371725	*32, *41, *69, *91, *119, *123, *138, *158
rs16947	*2, *4, *8, *11, *12, *14, *17, *19, *20, *21, *28, *29, *30, *31, *32, *34, *35, *36, *40, *41, *42, *45, *46, *51, *55, *56, *58, *59, *65, *69, *73, *84, *85, *91, *98, *102, *104, *105, *111, *114, *117, *121, *123, *125, *126, *128, *129, *133, *135, *136, *138, *141, *146, *148, *149, *150, *154, *155, *156, *157, *158, *159, *160, *162, *163, *165, *166, *171, *172, *175
rs1065852	*4, *10, *36, *37, *47, *49, *52, *54, *56, *64, *65, *69, *72, *87, *94, *95, *99, *100, *101, *114, *132, *142, *147, *150, *177
rs267608319	*31
rs769258	*35, *143, *172, *175
rs5030865	*8, *14, *114
rs1135840	*2, *4, *6, *8, *10, *11, *12, *14, *17, *19, *20, *21, *28, *29, *30, *31, *32, *35, *36, *37, *39, *40, *41, *42, *45, *46, *47, *49, *51, *52, *54, *55, *56, *58, *59, *64, *65, *69, *70, *72, *73, *83, *84, *85, *87, *88, *94, *95, *98, *99, *100, *101, *102, *103, *104, *105, *111, *114, *117, *121, *123, *125, *126, *128, *129, *132, *133, *135, *136, *138, *141, *142, *146, *147, *148, *149, *150, *154, *155, *156, *157, *158, *159, *160, *161, *162, *163, *164, *165, *166, *171, *172, *175, *177

The * within this figure stands for the alleles.

**Table 2:** Study participants demographics (N = 150).

Demographics	Descriptions	Number (n)	Percentage (%)
Age	<40	23	15.3
	40–49	88	58.7
	50–59	33	22
	>59	6	4.0
BMI	Underweight (≤18.4)	7	4.7
	Normal (15.5–25.0)	88	58.7
	Overweight (≥25.1)	49	32.7
	NA *	6	4.0
Stage	Stage I	36	24.0
	Stage IIA	48	32.0
	Stage IIB	16	10.7
	Stage IIIA	9	6.0
	Stage IIIB	11	7.3
	Stage IIIC	3	2.0
	Stage IV	12	8.0
	NA *	15	10.0
Menopausal status	Premenopausal	54	36.0
	Postmenopausal	96	64.0
Ethnicity	Ambon	2	1.3
	Batak	8	5.3
	Betawi	5	3.3
	Chinese	50	33.3
	Javanese	38	25.3
	Minangkabau	5	3.3
	Palembang	2	1.3
	Sunda	9	6.0
	Multiethnicity	25	16.9
	NA*	6	4.0

* NA: data not available.

**Table 3:** *CYP2D6* diplotype frequencies observed.

Diplotype	Phenotype	Number (n)	Frequency (%)
*10/*36	Intermediate Metabolizer	34	23.6
*1/*36	Normal Metabolizer	19	13.2
*2/*10	Normal Metabolizer	15	9.7
*1/*1	Normal Metabolizer	13	9.0
*2/*36	Normal Metabolizer	12	8.3
*1/*10	Normal Metabolizer	12	7.6
*10/*10	Normal Metabolizer	9	6.5
Others ^		36	22.2

^ Other diplotypes were observed with frequency less than 0.05, these diplotypes were *1/*2, *36/*41, *1/*41, *10/*41, *1/*5, *2/*2, *3/*36, *5/*10, *5/*41, *1/*3, *1/*4A, *14/*36, *2/*3, *2/*39, *2/*41, *36/*39, *4A/*10, and 3*/10*.

**Table 4:** Summary of metabolite levels at baseline in relation to *CYP2D6* metabolizer profiles.

*CYP2D6* Phenotype		Peripheral Whole Blood Concentration (ng/mL)			
		Tamoxifen	4-Hydroxy Tamoxifen	N-Desmethyl Tamoxifen	Endoxifen
Normal Metabolizers (n = 83)	SD	36.77	1.58	57.44	6.60
	Median	77.46	3.05	239.62	11.98
	Range	31.22–178.96	0.44–7.66	80.63–321.88	3.55–34.77
Intermediate Metabolizers and Poor Metabolizer (n = 65 + 2 = 67)	SD	36.26	2.68	56.90	4.35
	Median	81.58	3.22	239.11	8.33
	Range	14.22–210.39	0.54–9.31	77.61–340.41	3.17–22.97
*p*-value (*t*-test)		>0.05	>0.05	>0.05	<0.001 **

* IM and PM groups were merged for statistical analysis as there were only two participants belonging to PM group. ** Statistically significant *p*-value was observed among phenotype groups for endoxifen level difference.

**Table 5:** Summary of metabolite levels in IMs after dose adjustment compared to NMs at the baseline.

*CYP2D6* Phenotype		Peripheral Whole Blood Concentration (ng/mL)			
		Tamoxifen	4-Hydroxy Tamoxifen	N-Desmethyl Tamoxifen	Endoxifen
Normal Metabolizers at baseline (n = 83)	SD	36.77	1.58	57.44	6.60
	Median	77.46	3.05	239.62	11.98
	Range	31.22–178.96	0.44–7.66	80.63–321.88	3.55–34.77
Intermediate Metabolizers after adjustment (n = 30)	SD	48.77	22.79	76.99	4.54
	Median	161.95	6.29	324.90	13.54
	Range	80.59–254.96	3.34–12.99	236.80–501.90	7.68–23.37
*p*-value (*t*-test)		<0.001	<0.001	<0.001	>0.05 *

* There was no statistically significant difference in endoxifen levels of IM patients post dose adjustment compared to NM patients at the baseline (*p* > 0.05).

**Table 6:** Number and percentage of endocrine-related symptoms reported by study participants 8 weeks after post-test consultation with treating oncologists.

Symptoms	NM Participants Who Received 20 mg of Tamoxifen Daily (n = 31)		IM Participants Who Received 40 mg of Tamoxifen Daily (n = 26)		*p*-Value
	Participants Reported Side Effect (n)	Participants Reported Side Effect (%)	Participants Reported Side Effect (n)	Participants Reported Side Effect (%)	
Hot flashes	11	35.5	13	50.0	>0.05
Cold sweats	4	12.9	5	19.2	>0.05
Night sweats	9	29.0	7	26.9	>0.05
Vaginal discharge	12	38.7	11	42.3	>0.05
Vaginal itching/irritation	7	22.6	4	15.4	>0.05
Vaginal bleeding or spotting	5	16.1	6	23.1	>0.05
Vaginal dryness	10	32.3	3	11.5	>0.05
Pain or discomfort with intercourse *	16	51.6	1	3.9	<0.001 *
Lost interest in sex	20	64.5	4	15.4	<0.05 *
Weight gain	20	64.5	17	65.4	1
Lightheaded (dizzy)	11	35.5	9	34.6	1
Vomiting	2	6.5	1	3.9	1
Diarrhea	1	3.3	0	0.0	1
Headaches	9	29.0	14	53.9	>0.05
Bloating	12	38.7	12	46.2	>0.05
Breast sensitivity/tenderness	13	41.9	14	53.9	>0.05
Mood swings	23	74.2	17	65.4	>0.05
Irritable	18	58.1	16	61.5	>0.05
Pain in joints	21	67.7	13	50.0	>0.05

* Statistically significant *p*-values were observed.

## Data Availability

The data are available on request due to restrictions. The data presented in this study are available on request from the corresponding author. De-identified or anonymized datasets cannot be shared publicly due to ethical and legal reasons. Researchers were prohibited from sharing research data that contains sensitive patient information such as health history data as specified by Komisi Etik Penelitian Kedokteran Dan Kesehatan MRCCC Hospital, the Ethics Committee that has approved the study protocol. Permission to access research data is restricted to the corresponding authors only and data requests could be sent to Komisi Etik Penelitian Kedokteran Dan Kesehatan MRCCC Hospital (Tel.: +6221-2996-2888; Fax: +6221-2996-2778).

## Supplementary Materials

The following supporting information can be downloaded at: https://www.mdpi.com/article/10.3390/jpm15030093/s1, File S1: Initial Screening Form.

## Abbreviations

The following abbreviations are used in this manuscript:

ER	Estrogen receptor
*CYP2D6*	Cytochrome P450 2D6
NM	Normal metabolizer
UM	Ultrarapid metabolizer
IM	Intermediate metabolizer
PM	Poor metabolizer
RCT	Randomized controlled trial
BCCA	Breast Cancer Care Alliance
CPIC	Clinical Pharmacogenetics Implementation Consortium
gDNA	Genomic DNA
VAMS	Volumetric absorptive microsampling
MRM	Multiple reaction monitoring
OS	Overall survival
PFS	Progression-free survival
BMI	Body mass index
ITT	Intention-to-treat
FACT-ES	Functional Assessment of Cancer Therapy—Endocrine Subscale
AS	Activity score
