# Supplementary material for: CYP2D6 Genotyping for Optimization of Tamoxifen Therapy in Indonesian Women with ER+ Breast Cancer
Source: J Pers Med. 2025 Feb 28;15(3):93. doi: 10.3390/jpm15030093 (PMC11943653; doi:10.3390/jpm15030093)
Supplement: Supplementary file 1 [file jpm-15-00093-s001.zip › jpm-3440057-supplementary.pdf]

INITIAL SCREENING FORM

CYP2D6 Genotyping for Optimization of Tamoxifen Therapy in Indonesian Women with ER+ Breast Cancer

Principal Investigator **: Dr. dr. Samuel J. Haryono, Sp.B(K)Onk**

Name of Research Site : MRCCC Siloam Hospitals Semanggi

Patient ID : TMS / ☐☐☐ / ☐☐☐
site code / respondent number / initials (01-001-AAA)

Medical Record Number/*finger prick* : ☐☐☐☐☐.☐☐-☐☐-☐☐-☐☐

Buccal Swab Sample ID : ☐☐☐☐☐☐☐☐☐☐☐☐☐☐

Patient Data

1. Marital status : ☐ Single
   ☐ Married or living together
   ☐ Divorced/separated
   ☐ Widow/widower
2. Family history of cancer : ☐ Yes ☐ No
3. Children : ☐ Yes, number of children: ☐☐ ☐ No
4. Age of children : Child 1 → ☐☐ years
   Child 2 → ☐☐ years
   Child 3 → ☐☐ years
   Child 4 → ☐☐ years
5. Currently pregnant : ☐ Yes ☐ No
6. Currently breastfeeding : ☐ Yes ☐ No

7. Smoking habit : ☐ Yes ☐ No

8. Alcohol consumption (average volume 30 mL per serving):

☐ Never or <1 serving/month ☐ 5-6 servings/week

☐ 1-3 servings/month ☐ 1 serving/day

☐ 1 serving/week ☐ 2-3 servings/day

☐ 2-4 servings/week ☐ >4 servings/day

9. Meal frequency : ☐ 3 times a day

☐ 2 times a day

☐ >3 times a day

10. Fast food consumption : ☐ ≤Once a month

☐ Once a week

☐ 2-3 times a week

11. Exercise frequency : ☐ Every day

☐ 3-5 times a week

☐ 1-2 times a week

☐ Not regularly

12. Medical history (check all that apply):

☐ Diabetes mellitus ☐ Hypertension

☐ Arthritis ☐ Dyslipidemia

☐ Stroke ☐ Coronary heart disease

☐ Kidney disease ☐ Kidney stones

☐ Osteoporosis

☐ Cancer → ☐ Lung

☐ Gastrointestinal

☐ Liver

☐ Breast

☐ Kidney/bladder/testis

☐ Prostate

☐ Cervical

☐ Hematologic (blood)

☐ Brain/nervous system

☐ Skin

☐ Other (please specify): ____________________

☐ Other diseases, (please specify): ____

Inclusion Criteria

	Yes	No
Diagnosed with estrogen receptor-positive (ER+) breast cancer (early to late stage).		
Premenopausal patient.		
Has received tamoxifen therapy with a dose of 20 mg per day for at least 2 months.		
Completed and signed the informed consent form.<br>If "Yes," provide the date: ____________________________________		

Exclusion Criteria

	Yes	No
Has not received tamoxifen therapy for more than 2 months.		
Postmenopausal patient.		

Please fill in the table below according to the patient's indications.

**Parameter/Symptom**	**Results, Description, or Implications**
Breast abnormality location	☐ Right breast ☐ Left breast ☐ Bilateral
Tumor mass	☐ Positive ☐ Negative
Tumor location (breast quadrant)	
Tumor size (cm)	
Tumor consistency	
Tumor shape and borders	
Fixed to skin	☐ Yes ☐ No
Skin changes	☐ Redness ☐ Dimpling ☐ Edema/satellite nodules<br>☐ Peau d'orange ☐ Ulceration
Nipple changes	☐ Retraction ☐ Erosion ☐ Crusting ☐ Discharge
Axillary lymph nodes	Count:<br>Size:<br>Consistency:<br>☐ Fixed to each other or surrounding tissue
Infraclavicular lymph nodes	Count:<br>Size:<br>Consistency:<br>☐ Fixed to each other or surrounding tissue
Supraclavicular lymph nodes	Count:<br>Size:<br>Consistency:

	☐ Fixed to each other or surrounding tissue
Examination of the metastatic area, form, and patient complaints.	☐ Lung ☐ Liver ☐ Bone ☐ Brain<br>☐ Other: ________________________________
Immunohistochemistry test	☐ ER (+) ☐ ER (-)<br>☐ HER (+) ☐ HER (-)<br>Tumor markers:<br>CA 125 = ________________________________<br>CA 15-3 = ________________________________<br>CA 27.29 = ________________________________<br>CEA = ________________________________
Mammography examination	Performed?<br>☐ Yes ☐ No<br>If yes, please attach interpretation of mammography examination to this form.
Breast ultrasound examination	Performed?<br>☐ Yes ☐ No<br>If yes, please attach interpretation of breast ultrasound examination to this form.
MRI (Magnetic Resonance Imaging) examination	Performed?<br>☐ Yes ☐ No<br>If yes, please attach interpretation of MRI examination to this form.
CT-SCAN examination	Performed?<br>☐ Yes ☐ No<br>If yes, please attach interpretation of CT-Scan examination to this form.
Biopsy	Performed?<br>☐ Yes ☐ No<br>If yes, please attach biopsy results to this form.
Lab examination	Performed?<br>☐ Yes ☐ No<br>If yes, please attach interpretation of lab examination to this form.

Medications taken before this visit

**Medication Name**	**Unit (mg/tablet)**	**Dosage**	**Start Date**	**End Date** *leave blank if still taking the medication

Supplements or herbal medicines taken before this visit

**Supplement/Herbal Content**	**Unit (mg/tablet)**	**Dosage**	**Start Date**	**End Date** *leave blank if still taking the supplements/herbal medicines

Next follow-up date: ☐☐ (day) / ☐☐ (month) / ☐☐☐☐ (year)

Examiner’s Name:

Signature

____
